# Influence of Radiofrequency Surgery on Architecture of the Palatine Tonsils

**DOI:** 10.1155/2014/598257

**Published:** 2014-03-26

**Authors:** Jan Plzak, Pavla Macokova, Michal Zabrodsky, Jan Kastner, Petr Lastuvka, Jaromir Astl

**Affiliations:** ^1^Department of Otorhinolaryngology and Head and Neck Surgery, First Faculty of Medicine, Charles University and Faculty Hospital Motol, V Uvalu 84, 150 06 Prague 5, Czech Republic; ^2^Department of Pathology and Molecular Medicine, Second Faculty of Medicine, Charles University, V Uvalu 84, 150 06 Prague 5, Czech Republic

## Abstract

Radiofrequency surgery is a widely used modern technique for submucosal volume reduction of the tonsils. So far there is very limited information on morphologic changes in the human tonsils after radiofrequency surgery. We performed histopathological study of tonsillectomy specimens after previous bipolar radiofrequency induced thermotherapy (RFITT). A total of 83 patients underwent bipolar RFITT for hypertrophy of palatine tonsils. Tonsil volume reduction was measured by 3D ultrasonography. Five patients subsequently underwent tonsillectomy. Profound histopathological examination was performed to determine the effect of RFITT on tonsillar architecture. All tonsillectomy specimens showed the intact epithelium, intact germinal centers, normal vascularization, and no evidence of increased fibrosis. No microscopic morphological changes in tonsillectomy specimens after bipolar RFITT were observed. RFITT is an effective submucosal volume reduction procedure for treatment of hypertrophic palatine tonsils with no destructive effect on microscopic tonsillar architecture and hence most probably no functional adverse effect.

## 1. Introduction

Different surgical techniques are used to treat hypertrophy of the palatal tonsils. Beside classical tonsillectomy, which represents complete excision of the tonsils, subtotal removal of the tonsils preserving the tonsillar capsule and partially lymphoid tissue became very popular. Compared to classical tonsillectomy, the volume reduction is of lesser extent and a residual parenchyma remains. Nevertheless all so far used methods of tonsillotomy desolate tonsillar surface with consequent scarification of both mucosa and submucosa. A possible influence on mucosal function is still unresolved [1]. Therefore, mucosa sparing tonsillar reduction has been proposed. Only one surgical method completely spares the tonsillar mucosa, radiofrequency surgery. Nelson introduced radiofrequency volume reduction of the tonsils using Somnus generator with monopolar needle probe in 2000 [2]. Nowadays bipolar applicators (such as radiofrequency induced thermotherapy = RFITT system by Olympus Celon), which are anticipated to be safer than monopolar ones since there is a shorter track of electric current passing through the body between the bipolar electrodes, are widely employed. After an introduction of a needle-shaped applicator into the hypertrophic tissue, the high-frequency current (circa 500 Hz) acts in surrounding of the tip of the applicator. Exactly defined tissue volume is heated (depending on adjusted power and applicator shape). It causes necrosis, shrinkage, and volume reduction. The final volume reduction is reached after 6–8 weeks after the operation. Radiofrequency surgery is minimally invasive, almost bloodless, simple, fast, with low postoperative pain, and safe [3]. Surgery can be performed under local anesthesia as an outpatient procedure for well-cooperated patients.

RFITT reduces the volume of only a submucosal layer. The mucosa including a surface of deep crypts remains intact with supposedly no affection of its function as well as no alteration of tonsillar lymphoid function. So far there is very limited information on morphologic changes in the human tonsils after RFITT that could support this theory. Only Terk and Levine described one case of histopathological examination of tonsillectomy specimen after previous insufficient radiofrequency surgery using Ellman dual-frequency generator [4]. An absence of fibrosis and preservation of normal histologic architecture of the tonsils was noted. But they remarked that the case was their first attempt of radiofrequency volume reduction of tonsils so they delivered the limited amount of energy (2 sessions: 2 W power setting in four and five areas in each tonsil, resp.). They concluded that the application of energy for a longer period or at a higher power would have reduced the size of the tonsils even more in this case to prevent insufficient volume reduction. Delivery of the higher amount of energy, which is presently widely used, could more likely lead to any structural changes.

There are no data about microscopic effect in the tonsils after bipolar RFITT. Hence we collected tonsillectomy specimens after bipolar RFITT and performed profound histopathological examination comparing these cases to standard tonsillectomy ones.

## 2. Materials and Methods 

We treated 83 patients (19 males and 64 females; median age 27 years; age range 15–43 years) by RFITT of the tonsils from December 2005 till August 2010. Hypertrophy of the tonsils in patients with sleep-disordered breathing was indication for surgery in all cases. All procedures were performed in an outpatient clinic setting. Local anesthesia was achieved by surface mucosal application of 1% Xylocaine and by injection of approximately 5 mL 1% Mesocain plus 1 : 100.000 epinephrine directly into the tonsillar tissue. For bipolar RFITT we used CelonLab ENT device (Olympus, Teltow, Germany) with power set on 7 W plus Celon ProSleep applicator performing three to five punctures into each tonsil depending on its size in one session treatment. The tonsillar volume was calculated from ultrasound measurement of three tonsillar dimensions using an ovoid-shaped calculation model. We compared pretreatment results to three-month posttreatment ones.

Five patients with an insufficient self-subjective evaluation subsequently underwent traditional cold-steel bilateral tonsillectomy under general anesthesia 6–8 months after RFITT of the tonsils. Profound histopathological examination using haematoxylin and eosin staining on multiple (20) 5 micrometers sections was performed to determine the effect of RFITT on tonsillar architecture. All medical procedures were approved by the Institutional Review Board of the Faculty Hospital Motol.

## 3. Results 

The average volume of the tonsil before and after surgery was 4.4 mL (range 1.6–9.3 mL) and 2.6 mL (range 0.8–5.6), respectively. The average volume reduction of the tonsil in all treated patients was 38% = 1.8 mL (range 0%–71%). Preservation of the treatment results was monitored for 2–5 years after treatment, with no adverse after effects during this time period. Five patients were recommended for subsequent standard tonsillectomy under general anesthesia. All of them were treated by RFITT in four areas in the each tonsil. The cases were as follows: Case 1: woman, 23 years, 25% volume reduction of the left tonsil, and 32% volume reduction of the left tonsil; Case 2: woman, 28 years, 39% volume reduction of the left tonsil, and 34% volume reduction of the left tonsil; Case 3: woman, 27 years, 37% volume reduction of the left tonsil, and 45% volume reduction of the left tonsil; Case 4: man, 20 years, 19% volume reduction of the left tonsil, and 27% volume reduction of the left tonsil; Case 5: woman, 37 years, 43% volume reduction of the left tonsil, and 38% volume reduction of the left tonsil. The average volume reduction of the tonsil in these three patients was 34%. No complications (postoperative bleeding, inflammation) were observed during and after tonsillectomy as well as previous RFITT.

**Figure 1:**
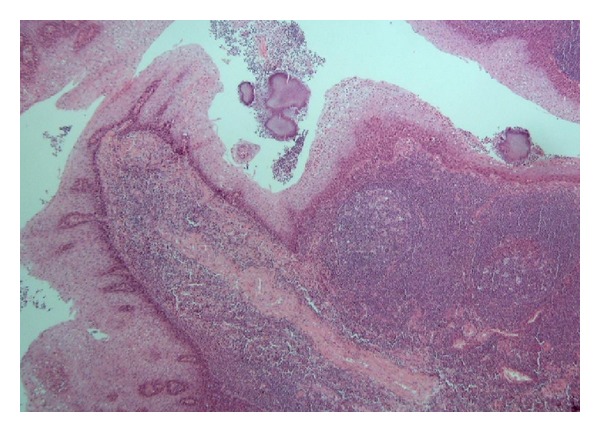
Tonsillectomy specimen 6 months after bipolar RFITT, haematoxylin-eosin. Normal structure of multilayer squamous epithelium and germinal centers, with no fibrosis and no hypervascularization present. Original magnification ×200.

Histopathological examination was analogical in all ten tonsillectomy specimens (Figure 1). Any neoplasia was excluded. The tonsillar epithelium preserved a normal structure of the multilayer squamous epithelium. Submucosally there were no signs of increased fibrosis corresponding to the scarification. Architecture of the lymphoid germinal centers was also normal as well as the extent and type of vascularization.

## 4. Discussion

Radiofrequency surgery is performed in different parts of the human body with diverse effects [5]. Although volume reduction impact has been generally proposed, it is not the result of treatment in each tissue. Relevant changes after radiofrequency surgery of the base of the tongue for obstructive sleep apnea could be observed neither for tongue volume or dimension nor for retrolingual space [6, 7]. The effects of radiofrequency surgery of the tongue base may more likely be a result of changes in upper airway collapsibility after scarring, postnecrotic fibrosis. Ciliated epithelium of the inferior nasal concha was not destroyed by radiofrequency reduction in patients with inferior nasal concha hypertrophy. Fibrosis of the subepithelial tissue was observed [8].

A different situation is in the tonsils. Significant macroscopic volume reduction verified by identical ultrasound volume measurement method was proved in our study in agreement with a work of Pfaar et al. [9]. The tonsil volume reduction after submucosal radiofrequency was as well proved by magnetic resonance imaging [10]. Despite any presumptions, there were no microscopic signs of chronic fibrosis and scarring or of germinal center remodeling several months after bipolar RFITT in our study. Why there is no microscopic effect? Palatine tonsils are included in the Waldeyer's lymphatic ring, the submucosal aggregation of lymphatic tissue with extensive immunogenic activity. We offer two hypotheses. (a) There is extremely activated “cleaning” immune reaction to RFITT injury caused by micro- and macrophages; (b) there is decreased proliferative component of healing reaction to RFITT injury caused by suppression of fibroblast proliferation and hypervascularization. RFITT-induced submucosal necrosis is probably completely removed without replacement by fibrous tissue [11].

Collection of more than ten tonsils would be better for evaluation of microscopic effect of RFITT. But bipolar RFITT of the tonsils gives so good result that “salvage” tonsillectomy is very rare. And there is no ethic justification to perform study with obligatory RFITT several months before planned tonsillectomy.

All five tonsillectomied cases did not deviate from other RFITT sufficiently treated ones concerning the indication, local finding, age, and peroperative and postoperative course. There are still no known predictive markers of response to bipolar RFITT of the tonsils.

Historical arguments against tonsillotomy such as an increased risk of chronic inflammation and higher incidence of peritonsillar abscess due to scarring of tonsillar tissue have never been confirmed [12]. In Reichel's study, comparing CO_2_-laser tonsillotomy and tonsillectomy, there was no statistically significant difference in preoperative serum anti-streptolysin-O antibody and immunoglobulin levels, C-reactive protein levels, and blood leukocyte counts between the two study groups. All specimens in both groups showed the histological picture of hyperplasia with no difference in the grades of hyperplasia. In all specimens signs of chronic inflammation could be detected. Even in patients with no history of recurrent tonsillitis, signs of chronic inflammation histologically were found in specimens after tonsillotomy. Clinical signs of recurrent tonsillitis after tonsillotomy were reported rarely. A low incidence of relapsing tonsillar hyperplasia after tonsillotomy should be expected. Reichel concluded that tonsillotomy could also be an option in some patients with mild recurrent tonsillitis [1]. Base on this conclusion, using RFITT in tonsillar hypertrophy with mild recurrent tonsillitis could be also considered [13].

Since bipolar RFITT of the tonsils causes neither epithelial nor submucosal scarring, we may assume that long-term followup should confirm the same effect for this up-to-date surgical technique. The real confirmation of no functional adverse effect of RFITT requires a longer followup (decades), which is not yet available because of short time of RFITT using tonsillar surgery worldwide since 2000 [2].

## 5. Conclusions

No microscopic morphological changes in tonsillectomy specimens after bipolar RFITT sustain the assumption of no functional adverse changes after this surgery. It is an effective submucosal volume reduction procedure for treatment of hypertrophic palatine tonsils with less postoperative pain, rare complications, and short recovery. The above mentioned facts are of high importance because a lot of patients potentially benefiting from bipolar RFITT of the tonsils are children. Bipolar RFITT of the tonsils may be performed in adults as an outpatient procedure.

## References

[1] Reichel O, Mayr D, Winterhoff J, de la Chaux R, Hagedorn H, Berghaus A (2007). Tonsillotomy or tonsillectomy?—a prospective study comparing histological and immunological findings in recurrent tonsillitis and tonsillar hyperplasia. *European Archives of Oto-Rhino-Laryngology*.

[2] Nelson LM (2000). Radiofrequency treatment for obstructive tonsillar hypertrophy. *Archives of Otolaryngology: Head and Neck Surgery*.

[3] Coticchia JM, Yun RD, Nelson L, Koempel J (2006). Temperature-controlled radiofrequency treatment of tonsillar hypertrophy for reduction of upper airway obstruction in pediatric patients. *Archives of Otolaryngology: Head and Neck Surgery*.

[4] Terk AR, Levine SB (2004). Radiofrequency volume tissue reduction of the tonsils: case report and histopathologic findings. *Ear, Nose and Throat Journal*.

[5] Ozlugedik S, Titiz A, Yilmaz YF, Tezer MS, Tezer A, Unal A (2007). Radiofrequency excision of a large postcricoid mucous cyst. *B-ENT*.

[6] Plzák J, Zábrodský M, Kastner J, Betka J, Klozar J (2013). Combined bipolar radiofrequency surgery of the tongue base and uvulopalatopharyngoplasty for obstructive sleep apnea. *Archives of Medical Science*.

[7] Stuck BA, Köpke J, Hörmann K (2005). Volumetric tissue reduction in radiofrequency surgery of the tongue base. *Otolaryngology: Head and Neck Surgery*.

[8] Sargon MF, Çelik HH, Uslu SS, Yücel ÖT, Denk CC, Ceylan A (2009). Histopathological examination of the effects of radiofrequency treatment on mucosa in patients with inferior nasal concha hypertrophy. *European Archives of Oto-Rhino-Laryngology*.

[9] Pfaar O, Spielhaupter M, Schirkowski A (2007). Treatment of hypertrophic palatine tonsils using bipolar radiofrequency-induced thermotherapy (RFITT). *Acta Oto-Laryngologica*.

[10] Nave H, Gebert A, Pabst R (2001). Morphology and immunology of the human palatine tonsil. *Anatomy and Embryology*.

[11] Bäck L, Tervahartiala P, Piilonen A, Mäkitie A, Ylikoski J (2009). Assessment of submucosal radiofrequency tonsil reduction by magnetic resonance imaging. *Journal of Otolaryngology: Head and Neck Surgery*.

[12] Unkel C, Lehnerdt G, Metz K, Jahnke K, Dost P (2004). Long-term results of laser-tonsillotomy in obstructive tonsillar hyperplasia. *Laryngo-Rhino-Otologie*.

[13] Stelter K, Ihrler S, Siedek V, Patscheider M, Braun T, Ledderose G (2012). 1-year follow-up after radiofrequency tonsillotomy and laser tonsillotomy in children: a prospective, double-blind, clinical study. *European Archives of Oto-Rhino-Laryngology*.

